# Effects of intermittently scanned continuous glucose monitoring on blood glucose control and the production of urinary ketone bodies in pregestational diabetes mellitus

**DOI:** 10.1186/s13098-021-00657-0

**Published:** 2021-04-09

**Authors:** Shu-ying Li, Hang Guo, Yi Zhang, Pei Li, Pei Zhou, Li-rong Sun, Jing Li, Li-ming Chen

**Affiliations:** 1 Department of Endocrinology, Tianjin Xiqing Hospital, Tianjin, China; 2 NHC Key Laboratory of Hormones and Development (Tianjin Medical University), Tianjin Key Laboratory of Metabolic Diseases, Tianjin Medical University Chu Hsien-I Memorial Hospital & Tianjin Institute of Endocrinology, Tianjin, 300134 China

**Keywords:** Pregestational diabetes mellitus, Intermittently scanned continuous glucose monitoring, Blood glucose control

## Abstract

**Objective:**

To investigate the effects of intermittently scanned continuous glucose monitoring (isCGM) on blood glucose control, clinical value of blood glucose monitoring and production of urinary ketone bodies in pregestational diabetes mellitus.

**Method:**

A total of 124 patients with pregestational diabetes mellitus at 12–14 weeks of gestation admitted to the gestational diabetes clinic of our hospital from December 2016 to December 2018 were selected and randomly divided into two groups. Sixty patients adopted self-monitoring of blood glucose (SMBG) were taken as the control group, and the other 64 patients adopted isCGM system by wearing the device for 14 days. Blood sugar control, glycosylated albumin level, ketone production in urine, the maximum and minimum of blood sugar value measured by different monitoring methods and their occurrence time were observed in the two groups.

**Result:**

(1) No statistically significant differences were found between the groups in terms of maternal age, gestational age at first visit, family history, duration of diabetes, education level, total insulin dose, chronic hypertension, abortion history, nulliparity, assisted reproductive technology, history of macrosomia childbirth, pre-pregnancy BMI, and overweight (%) at the first visit and hypoglycemia, (2) the value of Glycated Albumin was lower in the CGM group compared to the control group at 2ed weeks (14.6 ± 2.2 vs. 16.8 ± 2.7, p < 0.001). The women in the CGM group spent increased time in the recommended glucose control target range of 3.5–7.8 mmol/L (69 ± 10% vs. 62 ± 11%, p < 0.001) and reduced time above target compared with those in the control group at 2 weeks (25 ± 7% vs. 31 ± 8%, p < 0.001). In the second week of the study, the positive rate of urinary ketone body in isCGM group was lower than that in the control group (42 ± 5 vs. 54 ± 5, p < 0.001), and (3) the minimum blood glucose of 31.2% (20/64) cases in isCGM group appeared during 0:00–2:59 at night, and 26.6% (17/64) cases appeared during 3:00–5:59 at night. The minimum values of 40.0% (24/60) cases in the control group appeared within the 30 min before lunch, 23.3% (14/60) within the 30 min before breakfast, and 11.7% (7/60) within the 30 min before dinner. The cases of minimum of blood sugar before meals accounted for 75% of all the minimum values, and the cases of minimum at night only accounted for 8.3%.

**Conclusion:**

Intermittently scanned continuous glucose monitoring can reduce hyperglycemia exposure and ketone body formation in pregestational diabetes mellitus. In addition, isCGM is better than SMBG in detecting nocturnal hypoglycemia.

## Introduction

With the increasing prevalence of obesity, type 2 diabetes mellitus has become the most common disease among women of childbearing age [1]. The International Diabetes Federation (IDF) estimated that 21.3 million (16.2%) live births were delivered from women with hyperglycaemia in pregnancy (HIP) in 2017. Among them, gestational diabetes mellitus (GDM) accounted for 86.4%, pregestational diabetes mellitus (PGDM) accounted for 6.2%, and other types of diabetes (including type 1 and type 2 diabetes) found for the first time during pregnancy accounted for 7.4% [2]. Surveillance of chronic diseases and their risk factors in China in 2013 showed that the prevalence of adult diabetes was 10.9% [3], and in recent years, the incidence age of type 2 diabetes mellitus has gradually decreased. Thus, an increasingly larger number of women can be expected to have type 2 diabetes mellitus and its complications during pregnancy. In the mother, poor glycemic control during pregnancy can increase ketone body formation, cause abortion and fetal arrest, and lead to the need for a cesarean section due to fetal macrosomia [4]. In the infant, a major concern is hypoglycemia after birth. Poor glycemic control in pregnancy has even been shown to be related to the intelligence of offspring [5, 6]. Therefore, it is very important to control blood glucose during pregnancy.

Dynamic blood glucose monitoring is a technique that uses a glucose sensor to monitor the glucose concentration of subcutaneous interstitial fluid, thus indirectly reflecting the blood glucose level. Intermittently scanned continuous glucose monitoring (isCGM) can display the instant blood glucose as well as the blood glucose fluctuations from the entire day, and through an alarm system, it can notify the user about abnormal blood glucose levels at the current time or in the future. This increases the timeliness of insulin dose adjustment and hypoglycemia correction. In contrast, traditional SMBG can only reflect static and instantaneous blood glucose, and it is easy to omit concealed hypoglycemia and hyperglycemia, so it cannot comprehensively evaluate the changes of blood sugar [7]. Previous studies have primarily focused on the impact of dynamic blood glucose monitoring on blood glucose and pregnancy outcomes in pregnant patients with type 1 diabetes mellitus [8, 9], while the impact of this on blood glucose control and ketosis in pregnant patients with type 2 diabetes mellitus is rarely studied. Therefore, our study evaluates the outcomes of the two different monitoring methods, isCGM and SMBG, on blood glucose monitoring, blood glucose control, and ketosis in pregestational diabetes mellitus.

## Subjects and methods

### Subjects

A randomized controlled study was conducted on all pregnant patients at 12–14 weeks gestation with type 2 diabetes mellitus in our hospital from December 2016 to December 2018. Inclusion criteria was age between 21 and 40 years old, singleton gestation, and need for multiple insulin injections daily. The included participants also had to be skilled in using smartphones and willing to cooperate and follow-up regularly. Patients with multiple pregnancies and serious comorbid conditions were excluded. Additionally, patients with hyperemesis gravidarum were excluded. The purpose and significance of the study was explained to the subjects, their consent was obtained, and informed consent was signed. The study was approved by the Ethics Committee of Metabolic Disease Hospital of Tianjin Medical University (No. DXBYYhMEC2016-21).

### Methods

Patients were randomly divided into two groups using the Excel table random sampling method. The control group used SMBG to monitor blood glucose (n = 60); the isCGM group used intermittently scanned continuous glucose monitoring system to monitor blood glucose (n = 64).

Patients in the control group were monitored by SMBG. Blood glucose in capillaries was monitored eight times a day (before meals and 2 h after meals, before going to bed, and at midnight). Patients download the Dnurse App (Beijing Dnurse Technology Co., Ltd) in which they used the Outpatient mode. Fasting and post-prandial glucose self-measurements could be monitored using the Dnurse blood glucose meter (automatic data upload or manual data upload). Blood glucose data were compiled into tables and charts that were then uploaded and transmitted to the Doctor,s version of Dnurse App. IsCGM group patients were monitored by isCGM (FreeStyle Libre, Abbott). At the first visit, a professional diabetes nurse provided the patient with one-on-one equipment installation and system maintenance training. The glucose sensor was placed on the patient’s upper arm. The system automatically recorded the blood sugar value of the interstitial fluid every 15 min. In addition, glucose readings and glucose trends were obtained by scanning the sensor with a scanning detector. Patients wore the device for a total of 14 days, during which blood sugar values were uploaded through the diabetes management APP.

All patients personalized dietary guidance by an educational nurse during a patient’s first visit. This nurse calculates the amount of protein, fat, and carbohydrates the patient needs daily based on their weight and activity level, develops a training plan, helps patients choose appropriate aerobic exercise, and checks their diet and exercise performance on a return visit. The specialist established the outpatient medical service file for each patient in order to record data for each visit, including blood glucose and weight, which can help with disease assessment and treatment planning. Patients were expected to see the physician once a week for 3 weeks. At each visit, the doctor adjusted the dose of insulin according to patient's blood glucose. Every days during the study period, educational nurse provided APP online guidance by answering patients’ questions, such as questions related to diet, exercise, blood sugar monitoring, insulin injection. Patients’ blood glucose meters were required to regularly undergo comparisons with hospital laboratory blood glucose meters (Biosen C-Line Blood Glucose Analyzer, EKF, Germany) to correct for any testing error.

In both groups, urine ketone bodies were measured every morning and 4 pm, Urine ketone body test paper is distributed uniformly by educational nurse. The fasting target blood sugar range was 3.5–5.3 mmol/L, and the target range after a meal was 4.4–7.8 mmol/L.

### Statistical methods

Statistical analysis was performed using SPSS11.5 statistical software. The measurement data conforming to the normal distribution were expressed as ${\overline{\text{x}}}$ ± s. A *t*-test was used to compare the mean of the two groups, and a χ^2^ test was used to compare the constituent ratio of the two groups. A p-value < 0.05 was considered statistically significant.

## Results

### Control group included 60 patient and isCGM group included 64 patients

No statistically significant differences were found between the groups in terms of maternal age, gestational age at first visit, family history, duration of diabetes, education level, total insulin dose, chronic hypertension, abortion history, nulliparity, assisted reproductive technology, history of macrosomia childbirth, pre-pregnancy BMI, overweight at first visit and hypoglycemia. (*p* > 0.05) (see Table 1).

**Table 1:** Maternal baseline characteristics of the study groups

	Control group (n = 60)	isCGM group (n = 64)	*p-*value
Maternal age (years)	30.6 ± 3.1	31.2 ± 4.1	0.604
Gestational age at first visit (w)	9.4 ± 2.9	9.5 ± 2.5	0.726
Family history of DM, n (%)	21 (35.0)	23 (35.9)	0.732
Duration of diabetes (years)	5.2 ± 1.4	4.9 ± 1.2	0.728
University and above, n (%)	34 (56.7)	36 (56.2)	0.823
Severe hypoglycaemia in past year, n (%)	2 (3.3)	3 (4.7)	0.793
Total insulin dose (U/kg per day)	0.69 ± 0.14	0.65 ± 0.13	0.743
Chronic hypertension, n (%)	2 (3.3)	2 (3.1)	0.875
Abortion history, n (%)	11 (18.3)	12 (18.7)	0.842
Nulliparity, n (%)	35 (58.3)	39 (60.9)	0.732
ART, n (%)	2 (3.3)	3 (4.7)	0.531
History of macrosomia childbirth, n(%)	6 (10.0)	8 (12.5)	0.689
Pre-pregnancy BMI (kg/m^2^)	25.6 ± 2.9	25.7 ± 3.3	0.878
Overweight (%) at first visit 25 ≤ BMI < 28	35(58.3)	37(57.8)	0.741
Obesity(%) at first visit BMI ≥ 28	14 (23.3)	16 (25.0)	0.625

Severe hypoglycaemia was defined as an episode requiring third-party assistance

*ART* assisted reproductive technology, *BMI *(kg/m^2^) body mass index

### The glycemic control characteristics and ketonuria of the study groups are shown in Table 2

**Table 2:** The glycemic control characteristics and ketonuria of the study groups

	Control group (n = 60)	isCGM group (n = 64)	*p-*value
The value of GA at baseline (%)	18.6 ± 3.5	18.9 ± 3.6	0.852
The value of GA at 14th day (%)	16.8 ± 2.7	14.6 ± 2.2	< 0.001
The value of HbAlc at baseline (%)	7.1 ± 0.3	7.2 ± 0.4	0.796
Hypoglycaemia (%)	2 (3.3%)	3 (4.6%)	0.656
Time in target during the 1st week (3.5–7.8 mmol/L) (%)	54 ± 10	55 ± 11	0.703
Time in target during the 2ed week (3.5–7.8 mmol/L) (%)	62 ± 11	69 ± 10	< 0.001
Time > 7.8 mmol/L during the 1st week (%)	36 ± 7	35 ± 6	0.723
Time > 7.8 mmol/L during the 2ed week (%)	31 ± 8	25 ± 7	< 0.001
Time < 3.5 mmol/L during the 1st week (%)	8 ± 3	9 ± 4	0.689
Time < 3.5 mmol/L during the 2ed week (%)	6 ± 2	4 ± 3	0.092
Ketonuria positive during the 1st week (%)	55 ± 7	52 ± 6	0.698
Ketonuria positive during the 2ed week (%)	54 ± 5	42 ± 5	< 0.001

*GA* glycated albumin

The value of Glycated Albumin was lower in the isCGM group compared to the control group at 2ed weeks (14.6 ± 2.2 vs. 16.8 ± 2.7, p < 0.001). In the trial, the women in the isCGM group spent increased time in the recommended glucose control target range of 3.5–7.8 mmol/L and reduced time above target compared with those in the control group at 2 weeks. There was no significant difference between the two groups in baseline level and time less than the target value after 2 weeks. In the second week of the study, the positive rate of urinary ketone body in isCGM group was significantly lower than that in control group (42 ± 5 vs. 54 ± 5, p < 0.001) (see Table 2).

### Comparison of cases with maximum and minimum blood sugar values

In the control group, the minimum blood sugar values of 40.0% (24/60) cases appeared within the 30 min before lunch, 23.3% (14/60) within the 30 min before breakfast, and 11.7% (7/60) within the 30 min before dinner. The cases of minimum at night only accounted for 8.3%. Most of the maximum blood sugar appeared 2 h after three meals, totally accounting for 91.6% (55/60) of all cases. In isCGM group, the minimum blood sugar of 31.2% (20/64) cases appeared during 0:00–2:59 at night, 26.6% (17/64) cases appeared during 3:00–5:59 at night, and12.5% (8/64) cases appeared during 9:00–11:59 and 15:00–17:59. The maximum blood sugar of 40.6% (26/64) patients appeared at 6:00–8:59. Except for the periods of 2l:00–23:59, 0:00–2:59 and 3:00–5:59, where no maximum blood sugar appear, the maximum blood sugar was averagely distributed in other periods (see Table 3

**Table 3:** Comparison of cases with maximum and minimum blood sugar values of the study groups

Control group (n = 60)		isCGM group (n = 64)	
Minimum values at 0 clock, n (%)	5 (8.3)	Minimum values during 0:00–2:59, n (%)	20 (31.2)
Maximum values at 0 clock, n (%)	3 (5.0)	Maximum values during 0:00–2:59, n (%)	0 (0)
Minimum values before breakfast, n (%)	14 (23.3)	Minimum values during 3:00–5:59, n (%)	17 (26.6)
Maximum values before breakfast, n (%)	0 (0)	Maximum values during 3:00–5:59, n (%)	0 (0)
Minimum values after breakfast, n (%)	2 (3.3)	Minimum values during 6:00–8:59, n (%)	2 (3.12)
Maximum values after breakfast, n (%)	14 (23.3)	Maximum values during 6:00–8:59, n (%)	26 (40.6)
Minimum values before lunch, n (%)	24 (40.0)	Minimum values during 9:00–11:59, n (%)	8 (12.5)
Maximum values before lunch, n (%)	0 (0)	Maximum values during 9:00–11:59, n (%)	12 (18.7)
Minimum values after lunch, n (%)	2 (3.3)	Minimum values during 12:00–14:59, n (%)	3 (4.68)
Maximum values after lunch, n (%)	24 (40)	Maximum values during 12:00–14:59, n (%)	7 (11.0)
Minimum values before dinner, n (%)	7 (11.7)	Minimum values during 15:00–17:59, n (%)	8 (12.5)
Maximum values before dinner, n (%)	0 (0)	Maximum values during 15:00–17:59, n (%)	12 (18.7)
Minimum values after dinner, n (%)	1 (1.7)	Minimum values during 18:00–20:59, n (%)	3 (4.68)
Maximum values after dinner, n (%)	17 (28.3)	Maximum values during 18:00–20:59, n (%)	7 (11.0)
Minimum values before sleeping, n (%)	5 (8.3)	Minimum values during 21:00–23:59, n (%)	3 (4.68)
Maximum values before sleeping, n (%)	2 (3.3)	Maximum values during 21:00–23:59, n (%)	0 (0)

).

## Discussion

With improved understanding in the pathogenesis of diabetes mellitus, more attention is now being paid to the effects of fetal malnourishment (due to obesity during pregnancy, excess nutrition and gestational diabetes mellitus, etc.) on the fetus during pregnancy. Early pregnancy is a critical period for fetal growth and development. The “fetal origin” hypothesis for diabetes mellitus proposed by David Barker [10] suggests that a poor developmental environment during pregnancy could significantly increase the risk of a number of chronic metabolic diseases such as diabetes mellitus, obesity and cardiovascular disease (CVD) in adulthood.

Murphy et al. study [11] shows that hyperglycemia in pregnancy leads to an increased risk of adverse outcomes, including fetal hyperinsulinemia, which leads to accelerated fetal growth and development, causing macrosomia. This increases the risk of complications in childbirth, including shoulder dystocia and birth trauma. Additionally, insulin resistance during pregnancy causes significant fluctuation in blood glucose levels in the mother, which is also related to many adverse pregnancy outcomes [12, 13], Clearly, adequate blood glucose monitoring and maintenance during pregnancy is essential, particularly for mothers with DM [14, 15].

Common measures of blood glucose used in clinical practice are SMBG and hemoglobin A1c (HbAlc). SMBG can capture the blood glucose changes before meals, after meals, and at midnight for the course of 1 day, whereas HbAlc can estimate the average blood glucose level over the past 6–8 weeks. However, neither of these methods capture the dynamic changes of blood sugar throughout the day. The intermittently scanned CGM monitoring system can provide not only the current blood glucose value, but also the trend of blood glucose changes over the next 15 min. Users can achieve targeted treatment according to the blood glucose value and trend values. Additionally, the device will analyze and process the collected data, such as the blood glucose compliance rate, the frequency of hypoglycemia in each period, the average blood glucose in each period, and the average blood glucose during the working time of the sensor. These data can be exported by computer and be used to generate reports [16].

Denice et al. study [17] suggests that use of CGM during pregnancy is associated with improved neonatal outcomes, possibly due to reduced exposure of the fetus to maternal hyperglycemia. Our study obtained similar results to this, suggesting that continuous glucose monitoring can reduce the exposure to of the fetus to maternal hyperglycemia. The value of GA was lower in the CGM group compared to the control group at 2ed weeks (14.6 ± 2.2 vs. 16.8 ± 2.7, p < 0.001). The women in the CGM group spent increased time in the recommended glucose control target range of 3.5–7.8 mmol/L and reduced time above target compared with those in the control group at 2 weeks.

A large amount of fat in the body is mobilized to provide energy in pregnant patients with diabetes mellitus due to insulin deficiency and impaired blood glucose utilization, leading to increased ketones in the body. Ketonuria results when the increase in ketone body volume exceeds the tissue utilization capacity [18, 19]. In recent years, studies have found that the abnormal ketone body levels in pregnant women have a correlation with fetal malformation. Specifically, the accumulation of ketone bodies in pregnant women has adverse effects on the development of the fetal brain and nerves [20]. Therefore, it is important to detect the abnormal ketone body metabolism and act to correct the issue as early as possible. In our study, the number of “urine ketone body positive” patients in the isCGM group at week 2 was less than that in the control group. Quite possibly this is related to the reduction of hyperglycemia exposure to the fetus in pregnant women with isCGM. Hyperglycemic mothers should quickly take action to correct future hyperglycemia to avoid the fluctuation of blood glucose and subsequently avoid adverse effects on the baby.

Traditional methods are unable to fully assess patients’ blood glucose level, which may lead to wrong assessment of blood glucose and improper clinical treatment. Compared with traditional monitoring methods, isCGM can predict and adapt to the dynamic changes of patients' blood glucose throughout the day [21, 22]. Through the study of different monitoring methods, we found that compared with traditional SMBG monitoring methods, the lowest blood glucose in isCGM group patients mainly appeared at 0:00–5:59, suggesting that low blood glucose is more common at night. Using SMBG to monitor blood glucose found that the minimum blood glucose was mainly distributed before meals, and only 8.3% of the minimum blood glucose was at nighttime. This indicated that the presence of night low blood glucose could not be detected by SMBG alone. IsCGM monitoring of blood glucose can more accurately decipher hypoglycemia at night and hyperglycemia after breakfast, which can guide clinical medication to better control blood glucose and consequently reduce the occurrence of adverse pregnancy outcomes related to hyperglycemia.

Our study also has its limitations. First, the study time was limited, and there was a lack of long-term follow-up. Second, the level of blood ketones was not detected in our study, only urine ketones, and there are many other influencing factors on urine ketone body formation. Finally, we observed that women in the CGM group had more unplanned exposure than those in the control group, as patients often encountered problems when using CGM, which increased the contact between patients and medical staff than SMGB group. Third, Abbott’s FreeStyle Libre Flash Glucose Monitoring System is a limited precision in low glucose range. Hence, blood glucose below 4.0 mmol/L must be double-checked and confirmed by a glucometer.

## Conclusions

In conclusion, intermittently scanned CGM can comprehensively and dynamically evaluate blood glucose changes in real time. In our study, isCGM reduced the exposure of pregnant women to hyperglycemia, thus reduced the exposure of the fetus to hyperglycemia, and reduced the production of urinary ketone bodies. It was better at detecting nocturnal hypoglycemia.

## Data Availability

All data are freely available for scientific purpose.

## Abbreviations

isCGM: Intermittently scanned continuous glucose monitoring
SMBG: Self-monitoring of blood glucose
BMI: Body mass index
GDM: Gestational diabetes mellitus
PGDM: Pregestational diabetes mellitus
CVD: Cardiovascular disease
ART: Assisted reproductive technology
GA: Glycated albumin
